# Forecasting Zika Incidence in the 2016 Latin America Outbreak Combining Traditional Disease Surveillance with Search, Social Media, and News Report Data

**DOI:** 10.1371/journal.pntd.0005295

**Published:** 2017-01-13

**Authors:** Sarah F. McGough, John S. Brownstein, Jared B. Hawkins, Mauricio Santillana

**Affiliations:** 1 Harvard T.H. Chan School of Public Health, Boston, Massachusetts, United States of America; 2 Computational Health Informatics Program, Boston Children’s Hospital, Boston, Massachusetts, United States of America; 3 Computational Epidemiology Group, Division of Emergency Medicine, Boston Children’s Hospital, Boston, Massachusetts, United States of America; 4 Department of Pediatrics, Harvard Medical School, Boston, Massachusetts, United States of America; Institute for Disease Modeling, UNITED STATES

## Abstract

**Background:**

Over 400,000 people across the Americas are thought to have been infected with Zika virus as a consequence of the 2015–2016 Latin American outbreak. Official government-led case count data in Latin America are typically delayed by several weeks, making it difficult to track the disease in a timely manner. Thus, timely disease tracking systems are needed to design and assess interventions to mitigate disease transmission.

**Methodology/Principal Findings:**

We combined information from Zika-related Google searches, Twitter microblogs, and the HealthMap digital surveillance system with historical Zika suspected case counts to track and predict estimates of suspected weekly Zika cases during the 2015–2016 Latin American outbreak, up to three weeks ahead of the publication of official case data. We evaluated the predictive power of these data and used a dynamic multivariable approach to retrospectively produce predictions of weekly suspected cases for five countries: Colombia, El Salvador, Honduras, Venezuela, and Martinique. Models that combined Google (and Twitter data where available) with autoregressive information showed the best out-of-sample predictive accuracy for 1-week ahead predictions, whereas models that used only Google and Twitter typically performed best for 2- and 3-week ahead predictions.

**Significance:**

Given the significant delay in the release of official government-reported Zika case counts, we show that these Internet-based data streams can be used as timely and complementary ways to assess the dynamics of the outbreak.

## Author Summary

In the absence of access to real-time government-reported Zika case counts, we demonstrate the ability of Internet-based data sources to track the outbreak. Our model predictions fill a critical time-gap in existing Zika surveillance, given that early interventions and real-time surveillance are necessary to curb mosquito transmission. Official Zika case reports will likely continue to be delayed in their release; thus, it is important that health and government officials have access to real-time and future estimates of Zika activity in order to allocate resources according to potential changes in outbreak dynamics. The methodologies presented here may be expanded to any country–and perhaps finer spatial resolutions–to identify changes in Zika transmission for public health decision-makers.

## Introduction

The rapid spread of Zika virus has led to more than 400,000 suspected cases across the Americas since its introduction to Brazil in 2014, and has triggered alerts around the globe[1]. This event has led to diverse interventions and travel warnings to affected areas, underscoring the importance of proactive disease surveillance. While cases of sexual transmission of Zika virus have been documented[2], the virus is primarily transmitted through the bite of the *Aedes aegypti* mosquito and causes nonspecific flu-like symptoms and skin rashes[3,4]. Of particular concern is the possible link between Zika virus and neurological disorders such as microcephaly, a birth defect in which babies of infected pregnant women are born with abnormally small heads[5–8]. Over 1800 cases of Zika-related microcephaly and central nervous system disorders in newborns have been reported since the beginning of the epidemic, and the virus has spread to 70 countries globally[9]. In February 2016, the World Health Organization declared Zika a global public health emergency[10]. With no existing vaccinations or treatment for Zika infections, control of the *Aedes aegypti* mosquito is critical to curb the spread of the virus, as has been observed in dengue fever studies[11,12]. This requires continuous and up-to-date surveillance of cases to drive vector control interventions accordingly[13].

In countries with now autochthonous transmission, the surveillance of Zika infections is predominantly passive; cases are identified on the basis of hospitalizations and clinical symptom reports. The Pan American Health Organization (PAHO) currently streamlines reports from ministries of health, and reports weekly confirmed and suspected cases of Zika by country[14]. The release of these reports and those produced by the ministries, however, is typically delayed by three or more weeks due to systematic processing and data collection. As a consequence, the changing dynamics of Zika are frequently hard to be assessed in a timely manner, and thus, the availability of current data on Zika to the public and public health officials is limited.

In the past decade, the near real-time availability of novel and disparate internet-based data sources has motivated the development of complementary methodologies to track the incidence and spread of diseases. These approaches exploit near real-time information from internet search engines[15–18], news reports[19–21], clinician’s search engines[22], crowd-sourced participatory disease surveillance systems[23–25], Twitter microblogs[26–29], Electronic Health Records[30], and satellite images[31] to estimate the presence of a disease in a given location.

Some of the biases and errors observed when using these alternative data sources as individual indicators of disease incidence have been recently mitigated by using ensemble approaches that combine information from multiple data sources to produce a more robust disease estimate[32]. In parallel, multiple improvements have been proposed to disease tracking methodologies based on Google searches[33–38]. Finally, it has been shown that in the absence of information from traditional government-lead disease reporting, the combined use of news reports and Google’s search activity of the word “zika” in Colombia led to reasonable estimates of cumulative cases of Zika[20]. To the best of our knowledge, however, no attempts have been made to date to harness these and other digital data sources for near-real time weekly forecasting of Zika infections.

Here we assess the feasibility of using Zika-related Google search queries, Zika-related Twitter microblogs, and information from news reports collected by the web-based surveillance system HealthMap[16], in the prospective monitoring of Zika in five countries: Colombia, El Salvador, Honduras, Venezuela, and Martinique. In addition, we evaluate the ability of a collection of multivariable models that use information from these three data sources as input, to dynamically track and forecast the incidence of Zika virus up to 3 weeks ahead of the release of reports from PAHO, using multiple evaluation metrics.

## Methods

### Epidemiological data

We obtained weekly reports from the Pan American Health Organization (PAHO) that document the number of laboratory-confirmed and suspected cases of Zika in the Americas from the website (http://ais.paho.org/phip/viz/ed_zika_epicurve.asp) and from weekly epidemiological updates[39]. In the absence of this information, we obtained suspected and lab-confirmed Zika cases from epidemiological bulletins produced by the national Ministries of Health (MOH) of Colombia and Martinique[40,41]. Throughout the manuscript, we refer to these data as “official case count”. Due to the lack of robust diagnostic capabilities across the Americas and the estimated large number of asymptomatic cases[4,42], the present study focuses on predicting suspected Zika cases, which can be used as a proxy for potential hospital visits in each locality. This information could be useful for public health decision-makers when designing resource allocation plans. Under PAHO criteria, cases were classified as suspected if the patient presented a rash and two or more of the following symptoms: fever, conjunctivitis, arthralgia, myalgia, and peri-articular edema[43]. The time series of suspected cases spans the entire epidemic period of each country, beginning with the earliest reported cases through the last available epidemiologic week in the data (last accessed August 3, 2016). Data profiles for each country can be seen in Table 1.

**Table 1:** Data profile for countries.

	Colombia	Venezuela	Martinique	Honduras	El Salvador
Cumulative cases	92891	51043	33925	22705	11779
Number of search terms	26	15	8	11	12
Weeks of data	46	38	30	26	37
Week of first cases	8/9/15	10/11/15	12/27/15	12/13/15	9/20/15
Week of last accessible cases	7/10/16	6/26/16	7/17/16	5/29/16	5/29/15
Number of training weeks (G+T, AR / AGO+T / ARGO+TH)	20, 17	15, 12	12, 9	12, 9	17, 14

### Google search queries

The selection process of potentially useful search terms to track Zika avoided forward-looking bias and was performed via the Google Correlate and Google Trends tools (https://www.google.com/trends/correlate/;https://www.google.com/trends/). We identified the most highly correlated terms with the time evolution of Zika incidence in Colombia and Venezuela on Google Correlate within the time period of May 2015 to Jan 2016, and used Google Trends to identify search terms related to the term “Zika” for all five countries. The time window for the selection of these terms did not exceed the training period of each model. Because the output of Google Trends and Google Correlate consists of country-specific search terms, these are different for each country. All highly correlated terms to the query “Zika” were selected as model inputs without discrimination, including some potential misspellings of the disease such as “sika” and “sica”. We obtained weekly fractions of all identified Google search terms using the Google Trends website. The selected search terms were used as independent variables in the models and are shown in S1 Table.

### Twitter microblogs

We leveraged a custom script to access the free Twitter Public API to collect the maximum allowed number of tweets (up to 1% total Twitter volume) with any geographical coordinates. We then searched these tweets by country, using Twitter’s assigned country code and restricting to tweets in which this parameter was present, for the weekly volume of Twitter micro-blogs containing any of the words “Zika”, “microcephaly”, and “microcefalia”, but only Colombia and Venezuela had relevant Zika-related tweets, within the weeks of the epidemic outbreak, to merit the inclusion of Twitter data in our models. The fraction of tweets containing the Zika-related words when compared to the total number of tweets for each country was computed for every week and used as an independent variable in the models.

### HealthMap digital Zika surveillance

We obtained cumulative reported case counts of Zika virus disease in all countries via the HealthMap digital disease surveillance system (www.healthmap.org), which reports non-governmental media alerts of infections[16]. From these alerts, we calculated the weekly incidence of Zika infection for use as an independent variable in the models.

### Relationship between cases and Internet-based data

In order to assess whether the selected Google search terms, Twitter microblogs, and HealthMap-reported cases could be useful for weekly prediction of Zika incidence, we computed the Pearson’s correlation between each predictor and the official Zika case count, first for the training period of each country and later for the entire time series. In addition, we evaluated the autocorrelation of the signal itself (as lag-1, lag-2, and lag-3 terms). To determine the optimal linear relationship between the predictors and cases, we applied a series of simple transformations to these data and selected the transformation which produced the highest Pearson’s correlation. The results of this preliminary analysis was used for variable selection and to inform the dynamic transformation of variables process within the model, detailed below.

### Models

A collection of multivariable models, inspired by those introduced in the Flu prediction literature[30,37], were considered to estimate and forecast weekly suspected cases of Zika in the aforementioned five countries. These models used as input the weekly Google search frequencies of Zika-related terms, the fraction of Zika-related Twitter microblogs, cumulative Zika case counts as recorded by the HealthMap disease surveillance system, and the available historical official case count data at a given point in time. For consistency and comparability, all models (i) automatically select the most relevant search terms for prediction, (ii) incorporate new information on Zika cases as reports are released every week, and (iii) identify the best functional relationship between each input variable and the outcome variable, every week.

The selection of the most predictive input variables was performed using a penalized LASSO regression approach as described in[44]. While avoiding the use forward-looking information, we incorporated the most recently available information on Zika cases every week by dynamically expanding the time window of the training set of the models. Finally, at each week, we analyzed whether transforming each input variable would increase its correlation with the output variable. If this were the case, then the transformed value of the input variable producing the highest correlation with case data would be used as input for the model. As more epidemiological information becomes available, this dynamic transformation process allows the model to recursively recalibrate and incorporate changes in the relationships between the input variables and the case count information observed so far. The transformations we considered were not exhaustive and included the log(x), x^2^, and sqrt(x).

In addition to the models that used the aforementioned data streams as input, we built a collection of baseline models for comparison and context. We considered models that only used historical observation of Zika cases to predict cases on the subsequent weeks and models that incorporated information from these various data streams. Given the success of Google search terms in tracking other diseases as observed in [27,28], our models utilized Google search as a central predictor, and we explored the additions of Twitter and HealthMap data for the improvement of model predictions. Specifically, we considered (i) AR: a baseline lag-3 autoregressive model that used only Zika surveillance information from the prior 3 weeks to predict suspected cases, (ii) G+T: a model which used only Google search and Twitter (if available) data for prediction as introduced in[33] (iii) ARGO+T: a model which used autoregressive information and Google and Twitter (if available) data, adapted from[37], and (iv) ARGO+TH: a model which combined all data streams (Twitter if available, Google, HealthMap) with lag-3 autoregressive terms. For the two countries (Colombia and Venezuela) which had available Twitter data, we also constructed identical models (ii—iv) without this data source; that is, using Google and HealthMap data only. Our models are described by the following equation

$${\hat{y}}_{t}=\;\alpha_{t}+\;\overset{N}{\underset{i=1}{\sum }}\gamma_{i}y_{\left(t-i\right)}+\;\overset{K}{\underset{j=1}{\sum }}\beta_{j}X_{j,t}+\tau T_{t}+\eta H_{t}+\;\varepsilon_{t}\;\;\;\;\;\;\;\;\;\varepsilon_{t}\sim N\left(0,\sigma^{2}\right)$$

where we expand an autoregressive model of lag N with the inclusion of the fraction of Google search frequency X for each term j, the fraction of Twitter volume T, and HealthMap-reported cases H. As described in[37], autoregressive terms generally help maintain predictions within a reasonable range, while Google and Twitter information help the models to respond more rapidly to sudden changes in the dynamics. Due to the novelty of the Zika outbreak, stationarity was not used as a way to assess the appropriateness of using autoregressive models as a baseline; instead, we relied on the observed high autocorrelation of the signal with recent time lags of case counts and evidence of similar mosquito-borne outbreaks modeling approaches[45,46].

At each week, we used our models to generate predictions for 1, 2, and 3 weeks ahead of current time. To avoid future-looking bias in our predictions, forecasts were made using only the information available to each model at each week t; and for each time horizon our case count estimate was obtained using a different model. For instance, all models with autoregressive terms are restricted, in further week-ahead predictions, from accessing weeks of case data that have not yet occurred relative to week t. Thus, 3-week ahead (t+3) forecasts for model (i) were generated using only the lag-3 term (AR3) of official cases from 3 weeks prior to t+3: that is, using the observed cases available exactly at week t. 1-week ahead (t+1) forecasts for model (i), meanwhile, utilized all three AR1, AR2, and AR3 terms, which contain information on reported cases from the strictly observable weeks t, t-1, and t-2. In other words, data that would be unavailable in real-time for predictions—in our case, data on future infections—are excluded from each model. This same rule applies to models (iii) and (iv), which also include autoregressive information. Reflecting the delay in the release of case reporting, the models do access future weeks (relative to week t of case reporting) of Google searches, Twitter microblogs, and HealthMap-reported cases, since these digital streams are available closer to real-time than are official case data.

All models were trained through the same week in the time series and evaluated over the same time window, although the number of training weeks differed based on the information required in each model. Models containing autoregressive information began training 4 weeks into each epidemic, as opposed to training from the first week of reported cases, in order to necessarily inform the one-, two-, and three-week lag terms. A summary of dates and data used by country is shown in Table 1.

Models were fit as multiple generalized linear models with the glmnet package[47] in R v3.2.4[48], validated using k-fold cross validation, and evaluated for their out-of-sample predictive performance. For each model, we report three evaluation metrics: root mean square error (RMSE), the relative RMSE (rRMSE), and the Pearson correlation of predictions with observed cases, as detailed in[32]. Equations for each metric can be found in S1 Equations.

## Results

In order to evaluate the feasibility of using Zika-related Google searches, Twitter microblogs, HealthMap news reports, and historical official case counts to track Zika, we calculated the Pearson correlation between (a) the observed suspected case counts and each input variable, and (b) the observed suspected case counts and three transformations: log(x), x^2^, and sqrt(x), for each input variable. These transformations were observed to sometimes lead to better correlation values than the original raw variables for different time periods. S1 Fig displays in each country the best transformation of each input variable and suspected Zika case counts. From the multiple panels for each country, it can be seen that at least a subset of these (transformed) variables showed potential to be useful to track Zika. Indeed, correlations ranged from 0.93 to 0.56 in Colombia; 0.90 to 0.18 in Honduras; 0.39 to 0.29 in Venezuela; 0.69 to 0.13 in Martinique; and 0.92 to 0.41 in El Salvador. The lowest-correlation predictors tended to be the lag-3 autoregressive term, HealthMap-reported cases, and non-specific Google search terms like “Virus.”

**Fig 1:**
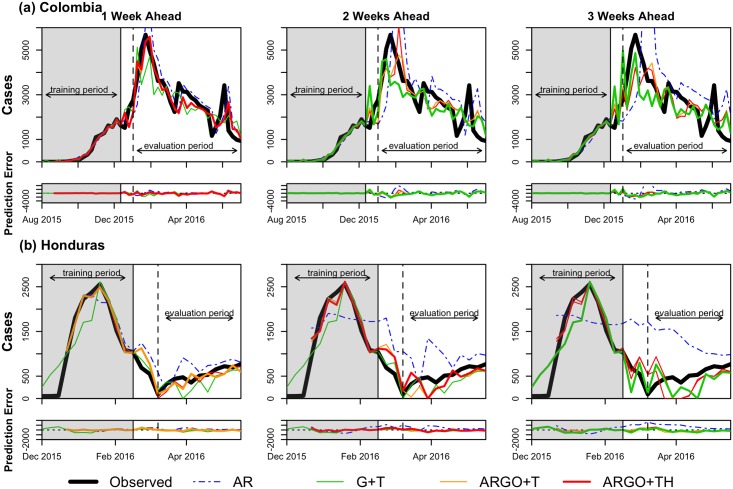
Prediction results for (a) Colombia and (b) Honduras. In each country, the weekly estimations of AR (dotted blue), G+T (green), ARGO+T (orange), and ARGO+TH (red) models are compared to the official case counts (black). Models include Twitter data where available (Colombia). The best model performance (lowest relative RMSE) in each time series by country is shown as a bolded line.

**Fig 2:**
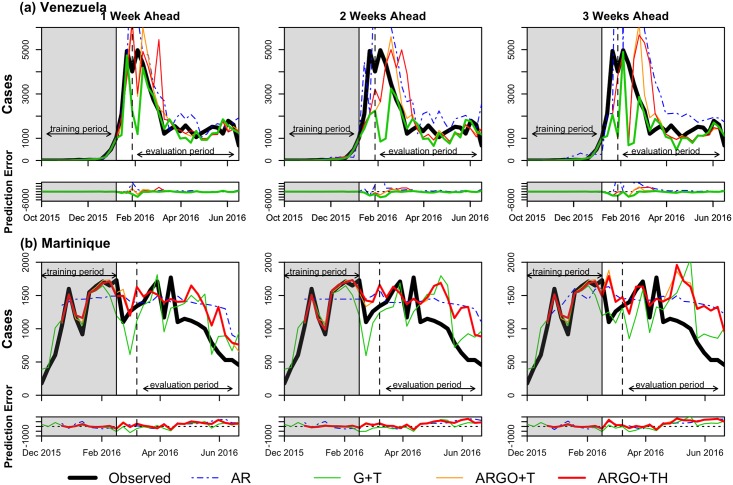
Prediction results for (a) Venezuela and (b) Martinique. In each country, the weekly estimations of AR (dotted blue), G+T (green), ARGO+T (orange), and ARGO+TH (red) models are compared to the official case counts (black). Models include Twitter data where available (Venezuela). The best model performance (lowest relative RMSE) in each time series by country is shown as a bolded line.

**Fig 3:**
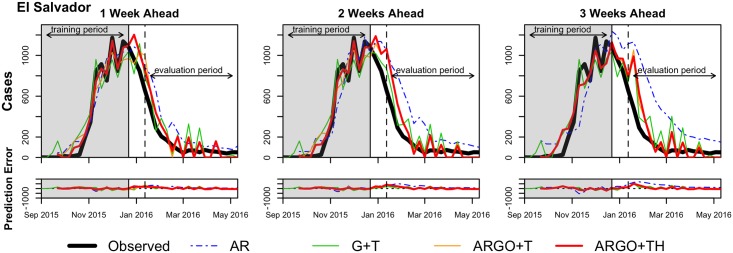
Prediction results for El Salvador. The weekly estimations of AR (dotted blue), G+T (green), ARGO+T (orange), and ARGO+TH (red) models are compared to the official case counts (black). The best model performance (lowest relative RMSE) in each time series is shown as a bolded line.

For each country, we produced out-of-sample predictions for the one, two, and three-week ahead time-horizons with the four models introduced in the previous section. We evaluated models according to the maximum number of data sources available, and thus assessed all models with Twitter data, where available (Colombia and Venezuela). In addition, we evaluated models with and without the inclusion of Twitter data. Plots comparing model predictions with the official Zika case count, by time horizon and country, are shown in Figs 1–3. Table 2 summarizes the out-of-sample predictive performance of the four models for each of the three week-ahead time horizons and for all countries, as captured by the three evaluation metrics. Note that while some model predictions showed high correlation values with official case counts, their predictions showed large discrepancies with the data. As a consequence, we relied on the relative RMSE (rRMSE) to establish the quality of model prediction given the short time span of the outbreaks. The rRMSE provides an estimate of the prediction error relative to the number of true cases observed in each week over the evaluation period, and, from our perspective, allows for better comparisons across models and time horizons. We henceforth judge model performance using this metric.

**Table 2:** RMSE, rRMSE, and Pearson's correlation coefficient (ρ) for 1-, 2-, and 3-week ahead out-of-sample predictions. Models include Twitter data where available (Colombia and Venezuela). The best fit metric for each week-ahead prediction is show in bold.

	**Colombia**								
**Model**	**1 week**			**2 week**			**3 week**		
	**RMSE**	**rRMSE**	**ρ**	**RMSE**	**rRMSE**	**ρ**	**RMSE**	**rRMSE**	**ρ**
AR	801.313	40.462	0.821	1484.018	66.829	0.539	2057.483	83.900	0.284
G+T	823.149	34.450	0.764	857.490	**37.300**	0.752	995.311	**41.903**	0.634
ARGO+T	621.673	30.076	0.870	**775.786**	39.583	**0.780**	914.643	44.233	0.679
ARGO+TH	**617.795**	**29.888**	**0.871**	848.968	40.153	0.731	**903.155**	42.440	**0.698**
	**Venezuela**								
	**1 week**			**2 week**			**3 week**		
	**RMSE**	**rRMSE**	**ρ**	**RMSE**	**rRMSE**	**ρ**	**RMSE**	**rRMSE**	**ρ**
AR	1665.733	68.542	0.822	4196.484	117.444	**0.834**	10349.050	259.699	**0.665**
G+T	972.937	**35.336**	0.626	1277.588	**39.813**	0.283	**1226.614**	**39.953**	0.475
ARGO+T	**892.063**	38.780	**0.831**	**927.343**	41.946	0.701	1372.884	48.249	0.486
ARGO+TH	1036.760	46.497	0.771	1148.229	67.028	0.626	1459.830	75.513	0.528
	**Martinique**								
	**1 week**			**2 week**			**3 week**		
	**RMSE**	**rRMSE**	**ρ**	**RMSE**	**rRMSE**	**ρ**	**RMSE**	**rRMSE**	**ρ**
AR	397.204	59.298	0.678	460.931	73.935	0.617	477.638	78.409	**0.744**
G+T	**302.038**	**40.123**	0.721	**376.475**	**47.758**	0.586	**450.635**	**53.835**	0.384
ARGO+T	336.375	42.998	**0.800**	425.005	61.420	0.701	510.691	73.822	0.492
ARGO+TH	342.577	44.923	0.799	424.417	61.382	**0.710**	506.310	73.423	0.482
	**Honduras**								
	**1 week**			**2 week**			**3 week**		
	**RMSE**	**rRMSE**	**ρ**	**RMSE**	**rRMSE**	**ρ**	**RMSE**	**rRMSE**	**ρ**
AR	262.701	167.009	0.546	538.930	330.114	-0.068	886.701	555.937	-0.903
G+T	213.788	53.909	0.675	222.045	51.993	**0.740**	**292.718**	**64.733**	**0.355**
ARGO+T	144.327	**30.436**	0.784	222.278	55.670	0.736	323.089	158.377	0.243
ARGO+TH	**132.675**	41.605	**0.853**	**203.616**	**51.874**	0.584	335.778	163.436	0.085
	**El Salvador**								
	**1 week**			**2 week**			**3 week**		
	**RMSE**	**rRMSE**	**ρ**	**RMSE**	**rRMSE**	**ρ**	**RMSE**	**rRMSE**	**ρ**
AR	159.185	126.486	**0.961**	261.119	234.615	0.929	379.797	350.656	0.888
G+T	120.979	166.901	0.881	**124.338**	152.882	0.911	180.282	187.945	0.855
ARGO+T	122.995	112.516	0.960	151.654	103.649	**0.976**	170.130	115.720	**0.923**
ARGO+TH	**100.318**	**110.603**	0.957	149.407	**103.143**	0.975	**166.552**	**113.459**	0.920

As seen in the evaluation metric values, no single model performed best across metrics, time horizons, and countries. Based on the rRMSE, models that combined Google (and Twitter data where available) with autoregressive information showed better predictive accuracy for 1-week ahead predictions. Meanwhile, models that only used Google (and Twitter where available) typically performed best for two and three-week ahead predictions.

The ARGO+T or ARGO+TH models outperformed all other models in 1-week forecasts for all countries with the exception of Venezuela and Martinique. In Venezuela and Martinique, the ARGO+T model (rRMSE = 38.8 and 43.0, respectively) slightly underperformed relative to the G+T model (rRMSE = 35.3 and 40.1, respectively), with a difference in rRMSE of about 3 percent points. In Colombia and El Salvador, the difference in rRMSE was less than 2% between the ARGO+TH and the ARGO+T models, with both models improving the rRMSE substantially compared to the G+T model.

In further week-ahead predictions, the Google and Twitter only (G+T) model outperformed models that also incorporated autoregressive information, exhibiting the lowest rRMSE in 3 of 5 countries for 2-week forecasts, and in 4 of 5 countries for 3-week forecasts.

Across models, prediction accuracy decreased as predictions were made further into the future, resulting in increases in rRMSE (and RMSE) and declines in model correlations across time horizons. Of all countries studied, Colombia had the best model performance in each week-ahead horizon for every model, with the exception of 3-week G+T forecasts; of all time horizons, the 1-week ahead predictions performed best in each country and model. In most cases, the autoregressive model over-predicted Zika incidence and underperformed all other models.

S2 Table shows the performance of additional versions of these models (i.e., the ARGO+T model with and without Twitter data). It can be seen that the inclusion of Twitter microblog data into our models improved or was comparable to (within 0.2 rRMSE) the performance of all models lacking Twitter data in Colombia (range of rRMSE reduction: -0.13, 1.6), and of the ARGO+T and ARGO+TH models in Venezuela (range of rRMSE reduction: 8.14, 125.1), for all time horizons. Conversely, incorporating HealthMap digital cases improved the rRMSE by no more than 3.8 points, or 7% (range: 0.06%, 6.8%) across models, time horizons, and countries, but worsened the rRMSE by up to 25.1 points, or 60% (range: 1.4%, 59.8%). The relative predictive power of each variable, as given by their standardized model coefficients, at each week in the out-of-sample predictions, is displayed in a collection of heatmaps in S2 Fig.

## Discussion

We have shown that Internet-based data sources can be used to track and forecast estimates of suspected weekly Zika cases, weeks ahead of the publication of official case counts. Models that rely exclusively on Google searches have among the lowest error (rRMSE) of all models, indicating that Google search terms alone have the potential to track Zika cases. The heatmaps shown in S2 Fig confirm that Google search terms have significant predictive power in most countries and time horizons.

In Colombia and Venezuela, where robust Twitter data were available, we found that Twitter improved predictions compared to models that lacked the data source. Meanwhile, though HealthMap news reports have been found to be good estimators of Zika cumulative incidence[20], the effect of incorporating HealthMap news reports into our models was marginal across countries and generally did not reduce prediction error in any of the weeks-ahead forecasts; where it did reduce prediction error, in El Salvador, it did by less than 2% compared to the next-best model lacking HealthMap data. We noted early evidence of HealthMap’s weak predictive power in its low correlation with official case counts, as shown in S1 Fig. Likewise, the heatmaps of S2 Fig reveal that news reports data generally had low influence in models after the first several weeks of out-of-sample predictions. We noted, however, in a post-hoc analysis, that news of Zika infections were 2–3 weeks delayed with respect to the time when cases had occurred. This fact suggests that in the absence of official case count reports, one may use (a potentially lagged version) of news reports to track Zika activity as found previously by[20]. In the future, we would expect to improve model predictions by incorporating HealthMap data lagged back in time by 2–3 weeks.

As seen in flu forecasting studies[32], the quality of predictions decreased as the time horizon of prediction increased. Specifically, for one-week predictions, we found that the model that uses Google (and Twitter where available) combined with autoregressive terms (the ARGO+T model) performs best in most countries, and its performance is better than or comparable to the equivalent model that lacks autoregressive information. Thus, the use of historical case information (autoregressive terms) improves predictions in the near future, a finding that has been documented in prior studies[26,30,37]. However, for 2–3 week-ahead predictions, models that use exclusively data from Google and Twitter (G+T), without autoregressive terms, perform best. This is likely because the 2–3 week old official case information is no longer crucial to refine the accuracy of predictions, and changes in Google search and Twitter activity better respond to fluctuations in Zika dynamics. Consequently, relying on historical case data becomes less useful in making predictions further into the future. This is also observed in the low relevance of lag terms in the 2- and 3-week heatmaps of all models (S2 Fig). Additionally, as automatically identified by our term selection methodology (LASSO), the predictive power of Google search terms is stronger in 1 week-ahead predictions than in 2 and 3 week-ahead predictions. This can be observed in the heatmaps shown in S2 Fig. This finding confirms the appropriateness of using a real-time hidden Markov process as a modeling framework, as discussed in [37]. From this perspective, people affected by Zika will search for Zika-related terms when affected by the virus or when they may suspect risk of exposure to it. This population search behavior suggests that monitoring search activity may help track disease incidence. The decreased relevance of search activity in 2 and 3 week-ahead predictions may suggest that autoregressive case count information may have a stronger role in future occurrences.

Our models improve upon prior methodologies[32,33,38] that use internet-based data sources to track flu by adding an internal dynamic variable transformation process to reassess the relationship of all input variables with the official Zika case count each week. Indeed, the heatmaps of variable coefficients show that model forecasts depended on an ensemble of terms whose predictive power changed magnitude and direction week by week. Given that Google queries were selected on the basis of their relationship to case data or to the term “Zika” exclusively in the training period, it is likely that these relationships change and perhaps even weaken in later weeks. We thus emphasize the importance the need for dynamic transformation of the input variables to recursively reassess these relationships and readjust predictors to their best linear fit with the data.

Some of the limitations of our approach include, for example, the inherent population biases of Internet search engines and Twitter microblog users. Internet searches patterns may also reflect media coverage and situational awareness that may not coincide with the dynamics of the disease being tracked. Also, different countries and locations frequently have distinct news reporting practices. Local media in regions with endemic mosquito-borne diseases may react differently to outbreaks than regions where these diseases are less frequent. Media attention thus has the potential to dramatically influence our weekly predictions. The dynamic reassessment of the predictive power of each input variable, via LASSO and the dynamic transformation approach discussed earlier, is built in our model to mitigate these events. Terms that may peak during a week of high media attention can be thrown out of the influence of the model for the subsequent week of prediction if their relationship with case count information has weakened. Only the terms with high predictive power are selected by the LASSO. In this way, our models are self-correcting. Nonetheless, we note that since our predictions rely largely on user search and media activity, our work is meaningful only in time periods when the population is aware of the disease; to this point, it has been demonstrated that Zika virus was introduced to Brazil and the Americas at least one year before the epidemic was recognized by health ministries and the public at large[49].

Another important consideration is the time lag between peaks in Zika virus incidence and microcephaly, of up to 5 months[50,51]. Our models capture search activity surrounding the Zika epidemic, and thus end up using search terms like “microcephaly” as input. These terms may be related to broader awareness of Zika activity. Given the estimated lag, however, evaluating microcephaly-related queries synchronously with cases has the potential to introduce a bias in the model. Further work must explore the effect of lagging these terms compared to our synchronous use of them.

As mentioned in the Methods section, Twitter data was not sufficient for use in the models for all countries. To improve upon this, future work could explore keyword queries that incorporate symptoms of Zika infection. In addition, to increase the total volume of tweets we plan to collect historical data based on these new query strings and explore ways to geocode the data ourselves, instead of relying on the current Twitter-generated subset of tweets with coordinate information.

Another challenge lies in the prediction of very low case numbers. In several weeks of the countries studied, official case counts of Zika fell below 50 suspected cases per week; this is very low relative to the thousands of cases experienced per week at the height of the epidemics. We observe that the quality of predictions decreases during time periods with low case numbers, and the model tends to under-predict cases. Our prediction approaches worked best in locations with highest Zika incidence, independently of Internet penetration. This tendency was also observed in the assessment of the Google Dengue Trends system in[38][45].

Limitations on the use of official suspected case counts from PAHO as our prediction goal include under-reporting. Indeed, Zika has been observed to be asymptomatic in at least 80% of infected persons[42]. As a consequence, our models likely underestimate the true number of Zika infections that exist, while reasonably estimating the actual number of suspected cases that seek medical attention. Unfortunately, no surveillance system has yet reported estimates of asymptomatic Zika infections, and it is unclear whether asymptomatic infections can result in the same consequences of birth and neurological defects as do symptomatic infections.

The predictions of our model should be compared to those of SIR-type models and epidemiologic models that evaluate Zika incidence in the context of important, known drivers of Zika, such as climate and ecological factors. In this paper, we explore whether digital data streams are viable estimators of Zika cases. In future inquiry, we believe that these methods could be incorporated into, and enhance, traditional epidemiologic methods to track the virus.

Given the need of early interventions to curb mosquito-borne disease transmission, our model predictions fill a critical time-gap in existing Zika surveillance since official case count reports will, most likely, continue being published multiple weeks after the occurrence of Zika cases. Moreover, access to real-time and likely future estimates of Zika activity provide an opportunity for health and government officials to allocate resources differently when potential changes in Zika dynamics are likely to occur, even ahead of official case documentation. The models presented here show promise to be expanded to any country at any time to track Zika cases and signal changes in transmission for public health decision-makers. Our models currently predict Zika activity at the country level, which we feel is useful for national decision-makers and surveillance purposes; however, our methodology can be extended to finer spatial units, such as the regional or municipal level. Performing predictions with higher spatial resolution will allow more targeted interventions and allocation of resources to the areas with the greatest projected burden of disease.

To produce these predictions in a publicly available and timely manner, we will work to create a website that displays Zika estimates for multiple countries continuously updated in real-time, similar to content published on www.healthmap.org/flutrends and www.healthmap.org/denguetrends.

## Supporting Information

S1 Equations Equations for (a) Root Mean Square Error (RMSE), (b) Relative Root Mean Square Error (rRMSE), and (c) Pearson Correlation. (TIFF)

S1 Table Google search terms used as input variables for each country. (XLS)

S2 Table Comparison of models in Colombia and Venezuela, with and without Twitter data. RMSE, rRMSE, and Pearson's correlation coefficient (ρ) are shown for 1-, 2-, and 3-week ahead out-of-sample predictive performance.
(XLS)

S1 Fig Correlation of digital predictors with official suspected Zika case counts in (a) Colombia, (b) Honduras, (c) Venezuela, (d) El Salvador, and (e) Martinique. The transformation that produced the highest correlation with Zika cases for each variable is shown in each plot. Data points from weeks within the training period are distinguished in red.
(PDF)

S2 Fig Heatmaps showing the relative influence (positive: red; negative: blue) of all input variables on predictions of Zika cases in (a) Colombia, (b) Honduras, (c) Venezuela, (d) El Salvador, and (e) Martinique. (PDF)
