# Supplementary figures and images for: Forecasting Zika Incidence in the 2016 Latin America Outbreak Combining Traditional Disease Surveillance with Search, Social Media, and News Report Data
Source: PLoS Negl Trop Dis. 2017 Jan 13;11(1):e0005295. doi: 10.1371/journal.pntd.0005295 (PMC5268704; doi:10.1371/journal.pntd.0005295)

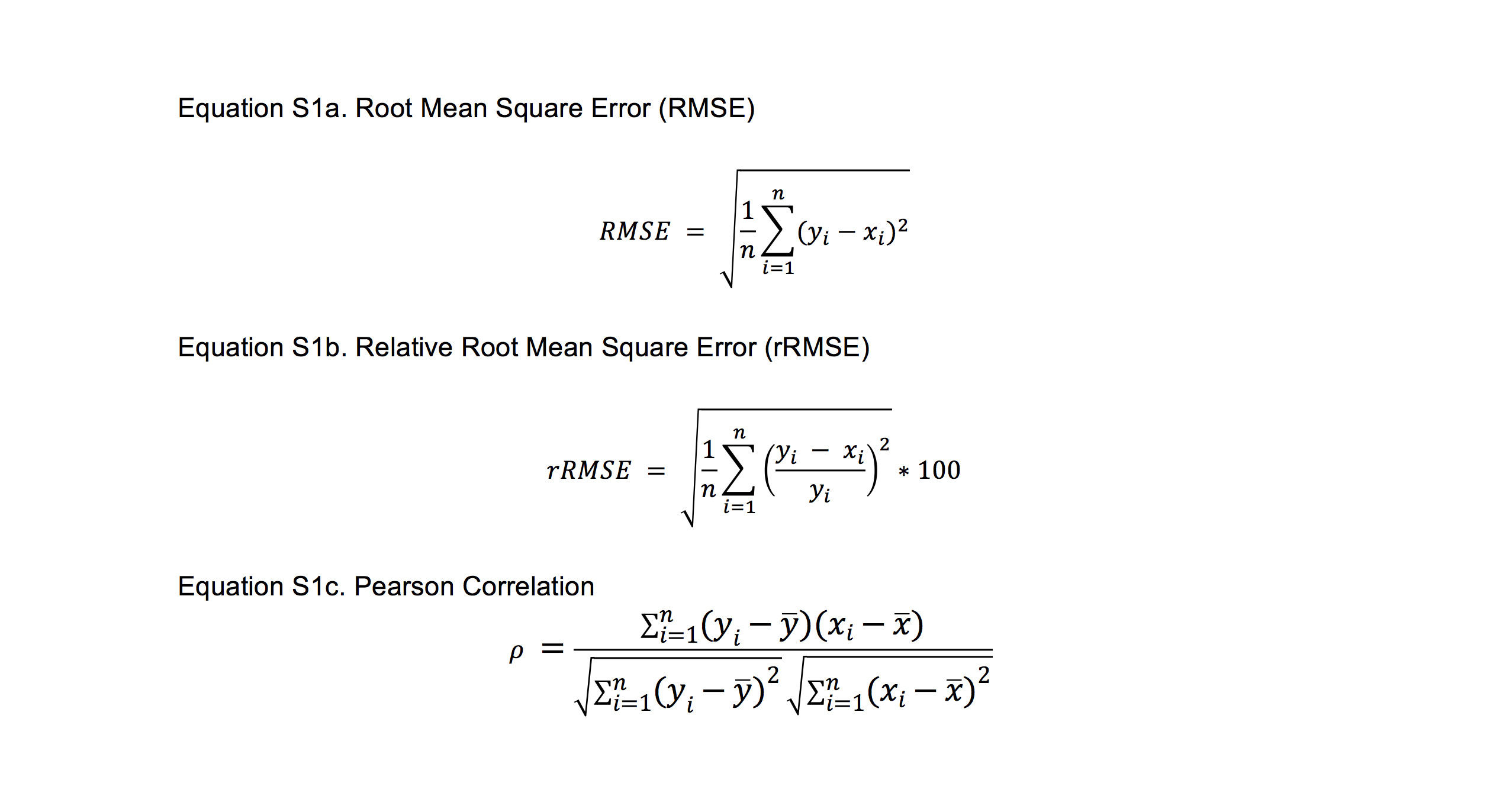

Supplement: S1 Equations — (TIFF) [file pntd.0005295.s001.tiff]

A

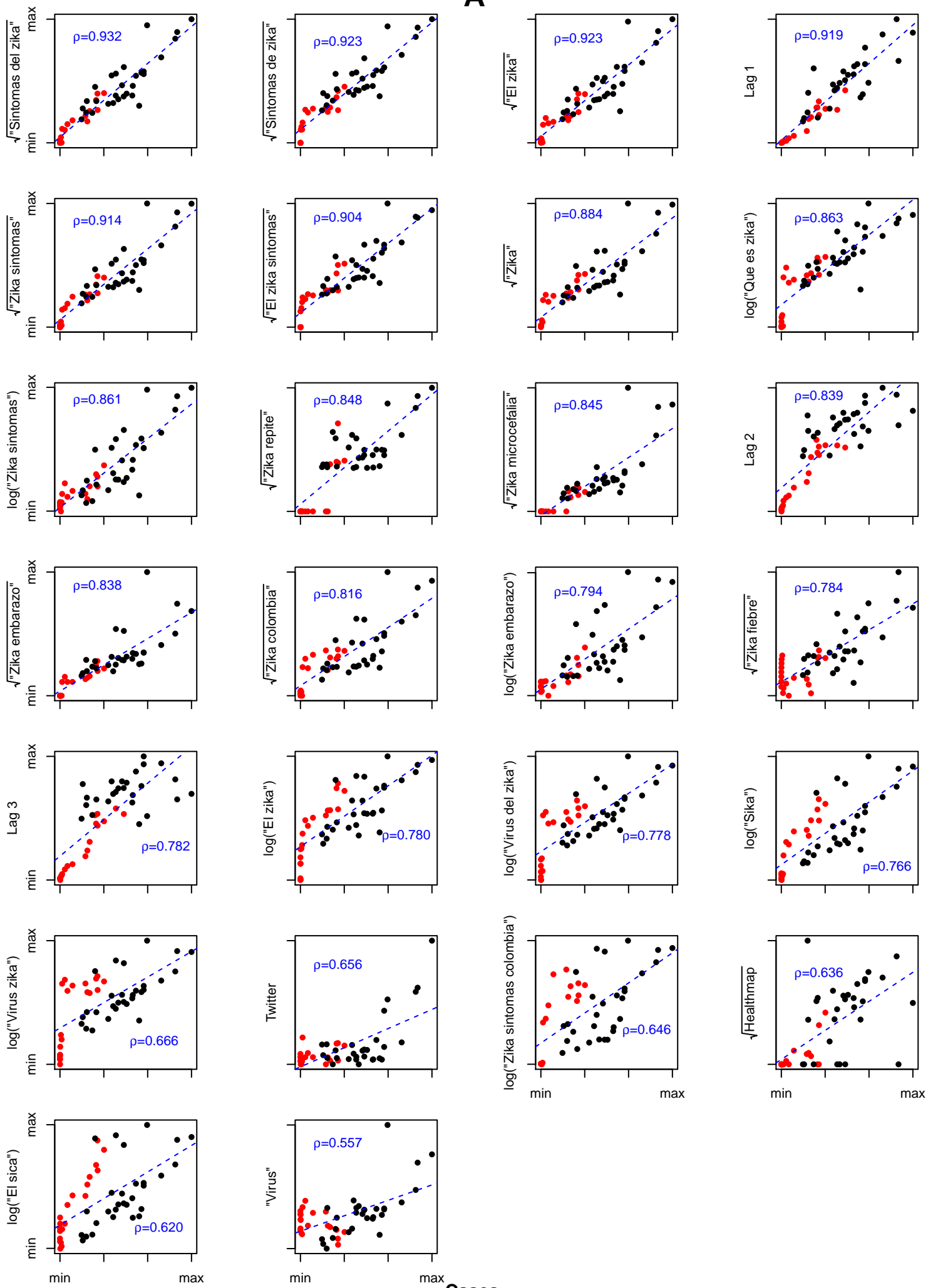

**B**

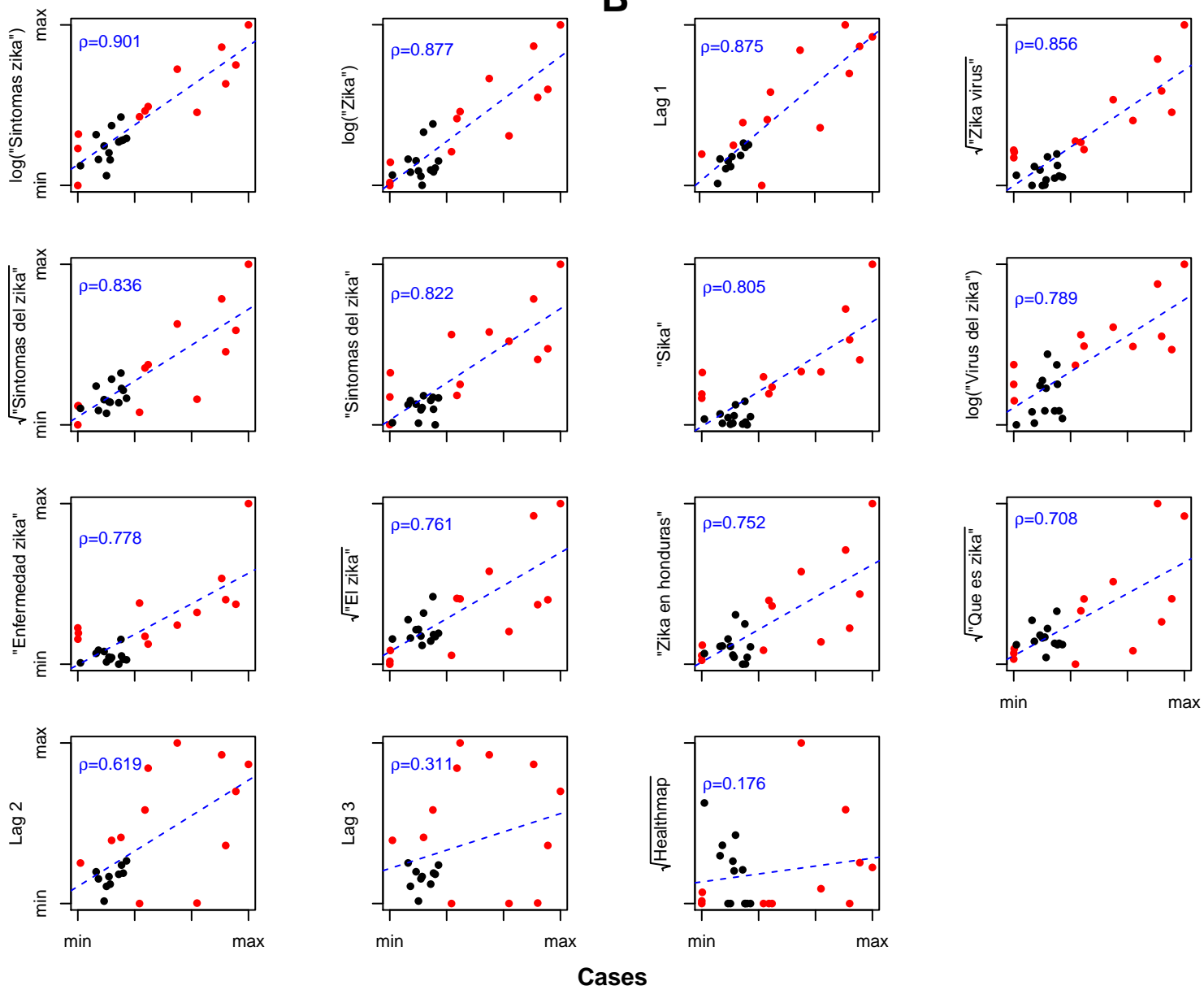

C

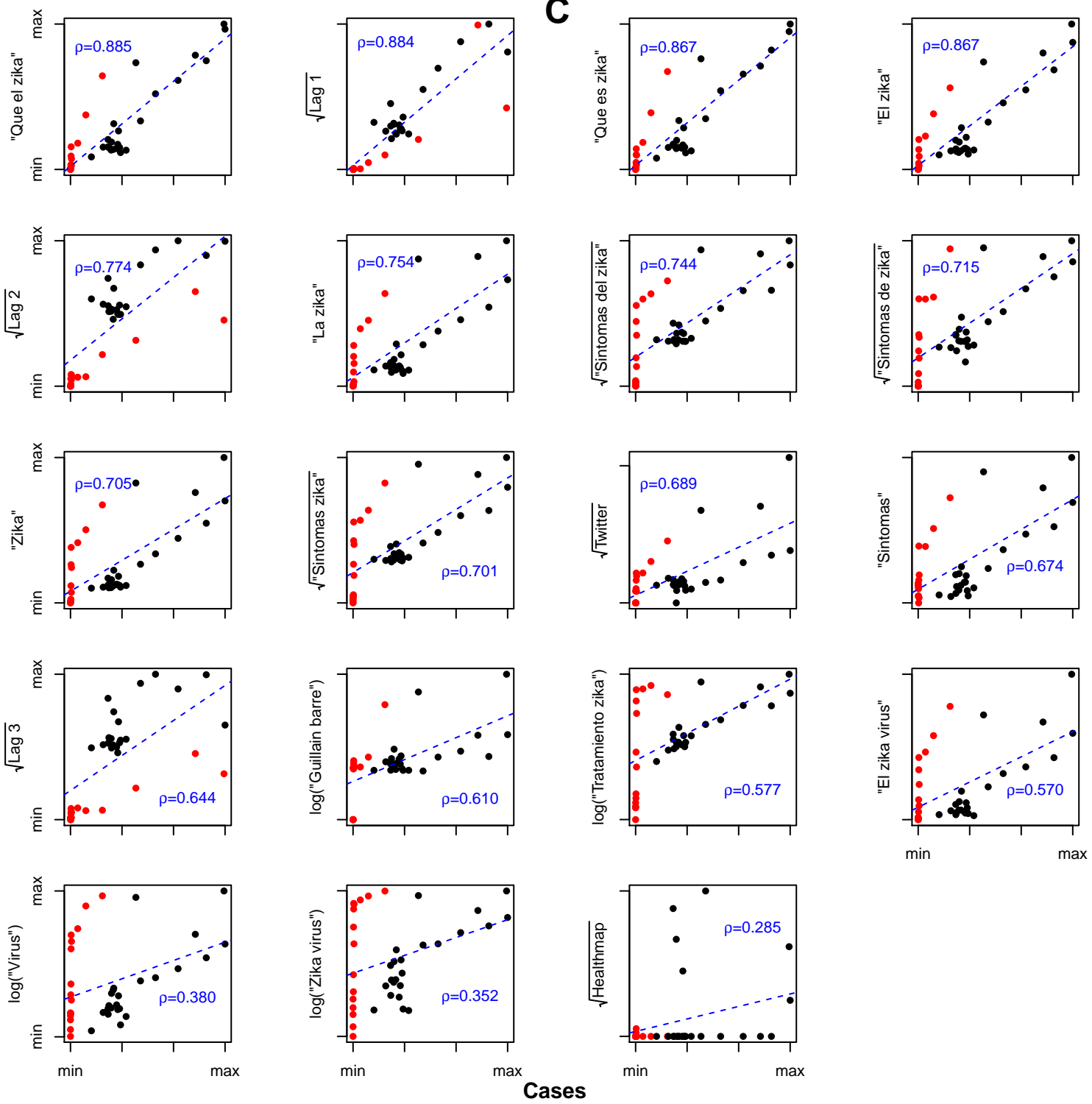

Cases

D

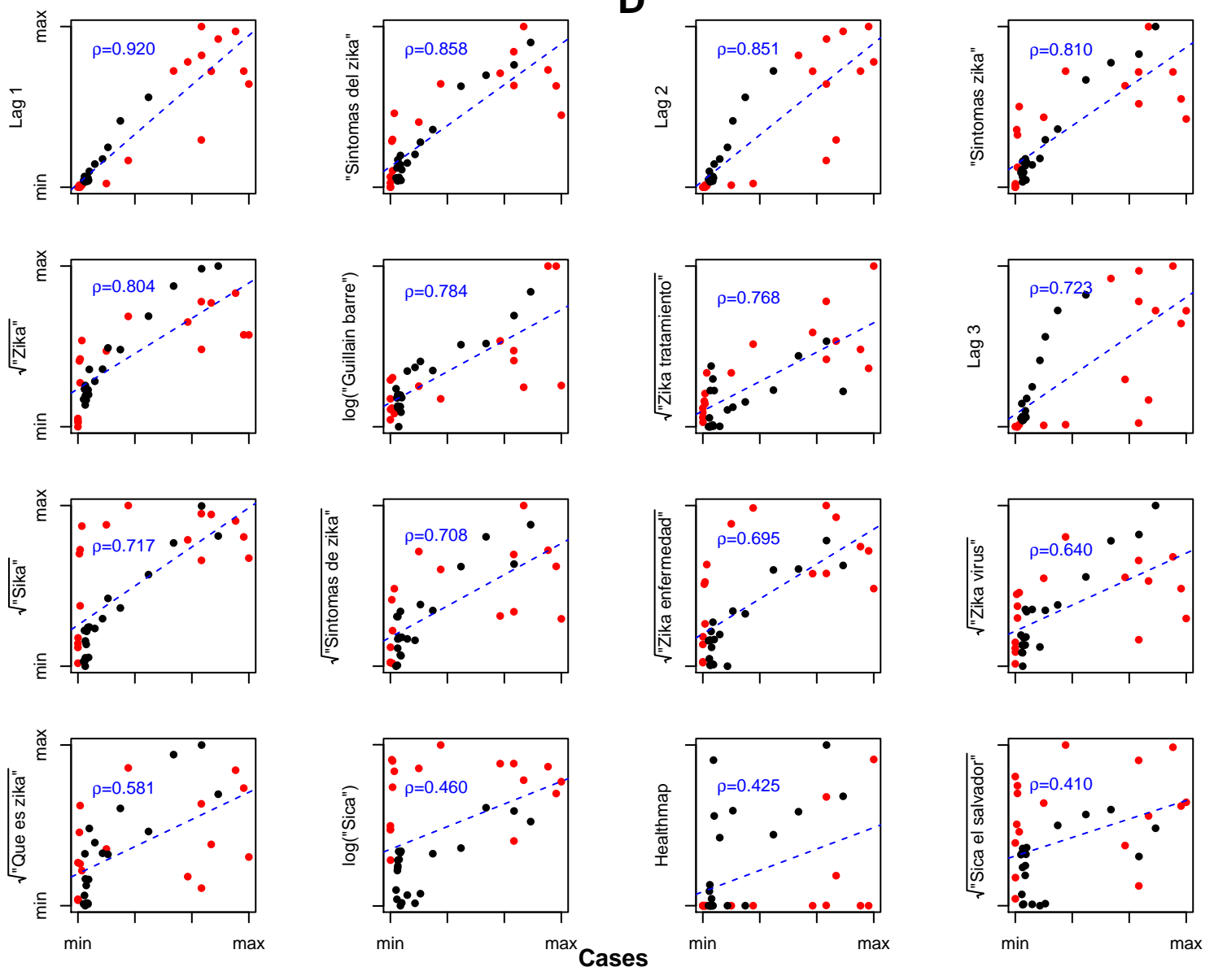

E

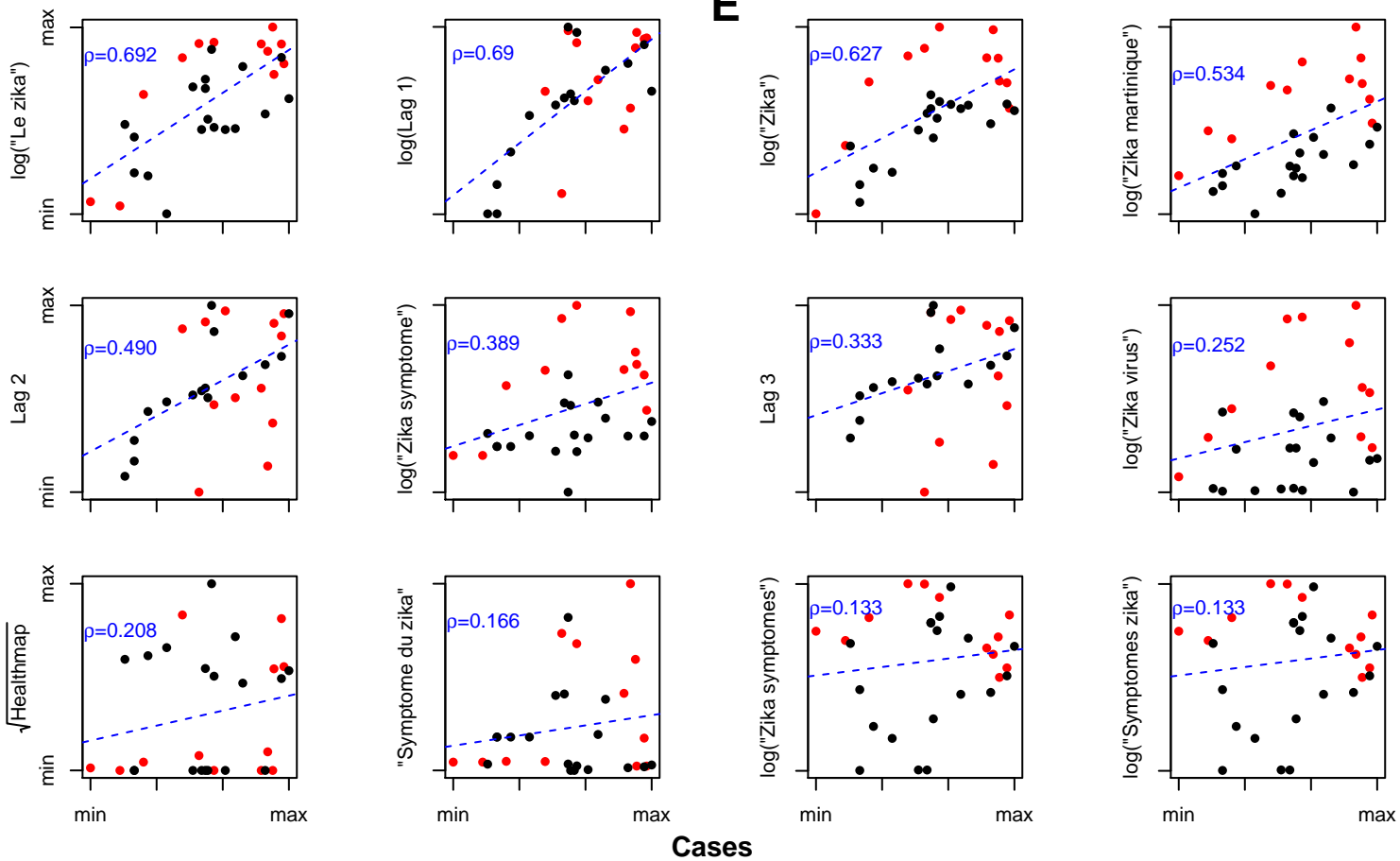

Supplement: S1 Fig — The transformation that produced the highest correlation with Zika cases for each variable is shown in each plot. Data points from weeks within the training period are distinguished in red. (PDF) [file pntd.0005295.s004.pdf]

### A. 1 Week Ahead

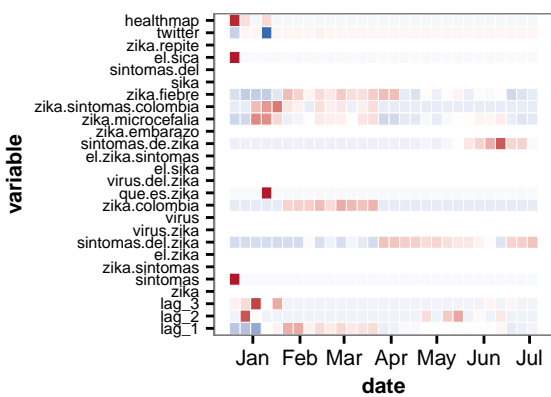

## 2 Weeks Ahead

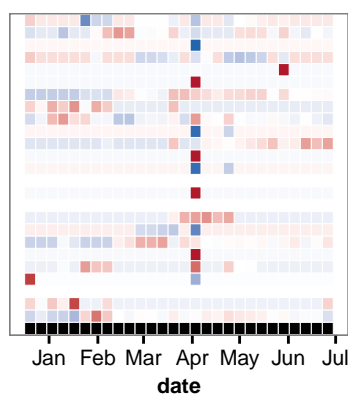

### 3 Weeks Ahead

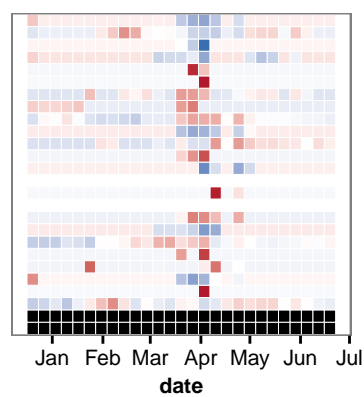

**B.**

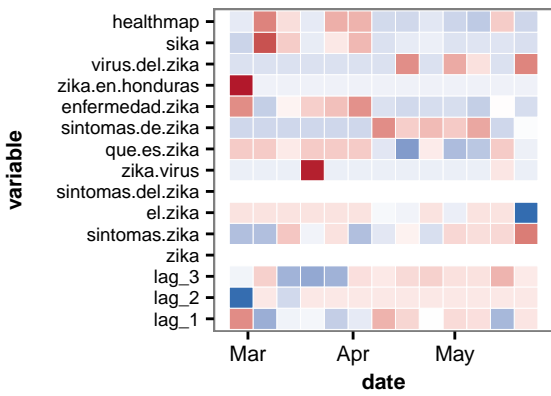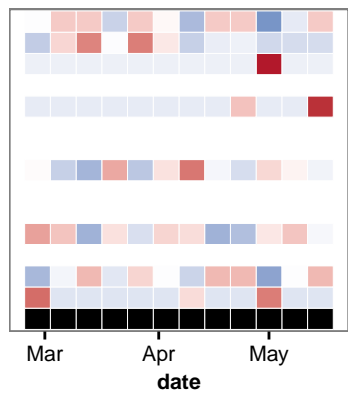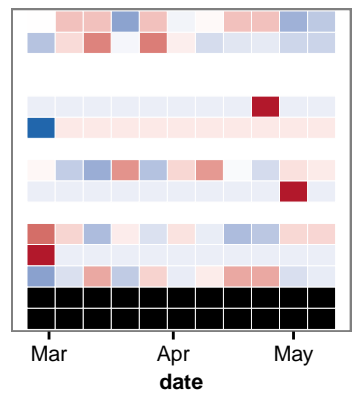

**C.**

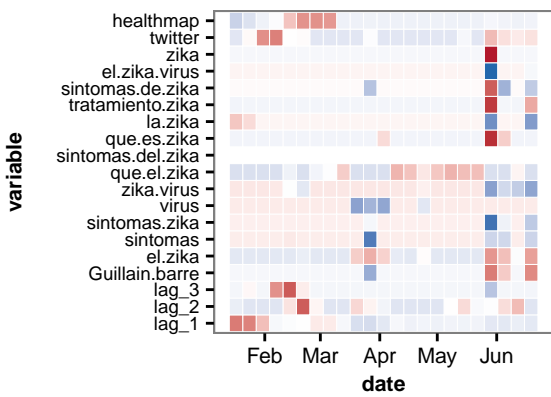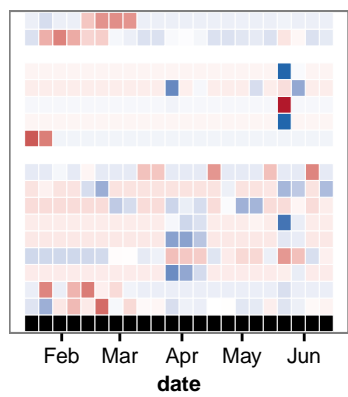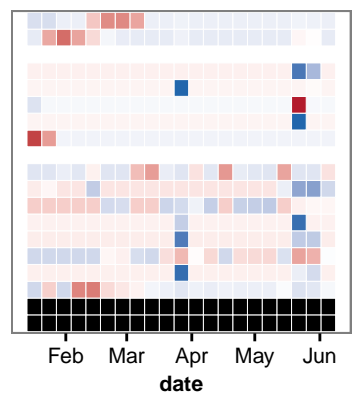

**D.**

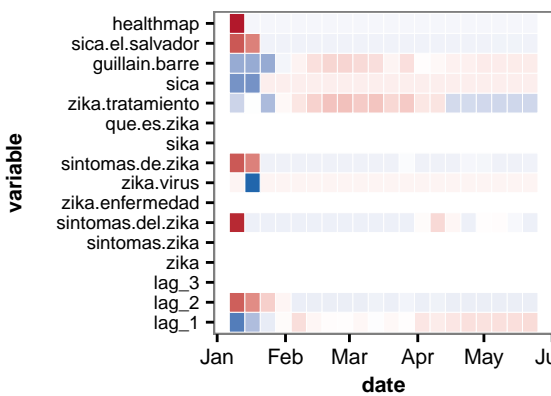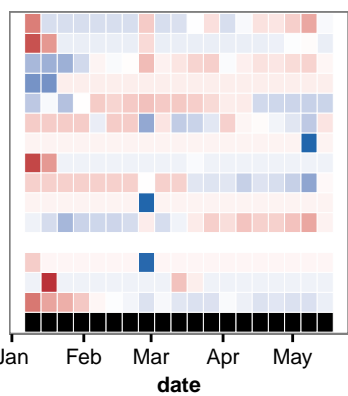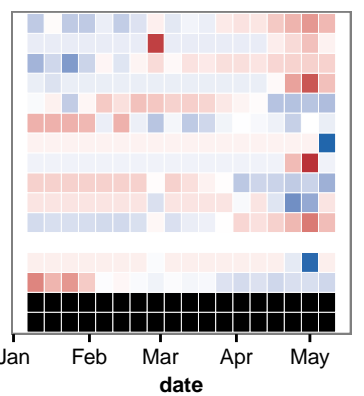

**E.**

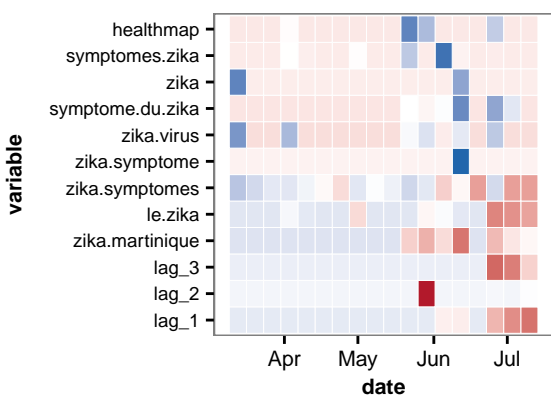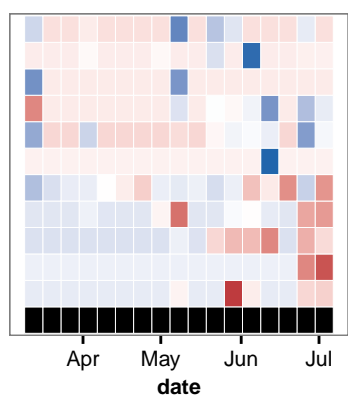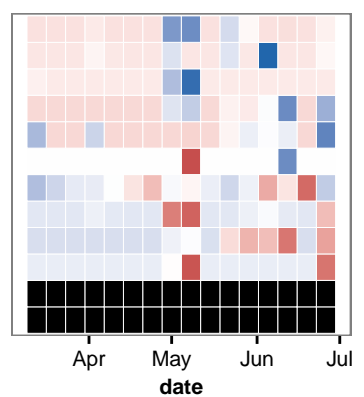

Supplement: S2 Fig — (PDF) [file pntd.0005295.s005.pdf]
